# In vitro and in vivo stability of a highly efficient long-acting cocaine hydrolase

**DOI:** 10.1038/s41598-024-61646-7

**Published:** 2024-05-13

**Authors:** Linyue Shang, Huimei Wei, Jing Deng, Madeline J. Stewart, Johnathan E. LeSaint, Annet Kyomuhangi, Shawn Park, Elise C. Maul, Chang-Guo Zhan, Fang Zheng

**Affiliations:** 1 Molecular Modeling and Biopharmaceutical Center, College of Pharmacy, University of Kentucky, 789 South Limestone Street, Lexington, KY 40536 USA; 2 Department of Pharmaceutical Sciences, College of Pharmacy, University of Kentucky, 789 South Limestone Street, Lexington, KY 40536 USA

**Keywords:** Cocaine abuse, Cocaine hydrolase, Enzyme therapy, Protein stability, Pharmacokinetics, Enzymes, Recombinant protein therapy

## Abstract

It is recognized as a promising therapeutic strategy for cocaine use disorder to develop an efficient enzyme which can rapidly convert cocaine to physiologically inactive metabolites. We have designed and discovered a series of highly efficient cocaine hydrolases, including CocH5-Fc(M6) which is the currently known as the most efficient cocaine hydrolase with both the highest catalytic activity against (−)-cocaine and the longest biological half-life in rats. In the present study, we characterized the time courses of protein appearance, pH, structural integrity, and catalytic activity against cocaine in vitro and in vivo of a CocH5-Fc(M6) bulk drug substance produced in a bioreactor for its in vitro and in vivo stability after long-time storage under various temperatures (− 80, − 20, 4, 25, or 37 °C). Specifically, all the tested properties of the CocH5-Fc(M6) protein did not significantly change after the protein was stored at any of four temperatures including − 80, − 20, 4, and 25 °C for ~ 18 months. In comparison, at 37 °C, the protein was less stable, with a half-life of ~ 82 days for cocaine hydrolysis activity. Additionally, the in vivo studies further confirmed the linear elimination PK profile of CocH5-Fc(M6) with an elimination half-life of ~ 9 days. All the in vitro and in vivo data on the efficacy and stability of CocH5-Fc(M6) have consistently demonstrated that CocH5-Fc(M6) has the desired in vitro and in vivo stability as a promising therapeutic candidate for treatment of cocaine use disorder.

## Introduction

Cocaine is a widely abused drug and its growing impact on public health is of great concern. There is no Food and Drug Administration (FDA)-approved medication specific for cocaine overdose or dependence^1,2^. Developing an effective medication for the treatment of cocaine overdose and dependence continues to be a research priority. In principle, a truly effective pharmacological treatment for cocaine use disorder should be able to effectively attenuate the pharmacological effects of cocaine without affecting the normal functions of the brain receptors and transporters. For the treatment of substance use disorders in general, traditional pharmacological approaches use small-molecule compounds as agonists/antagonists directly or indirectly blocking the neuropharmacological action of the drug^3–5^. However, it is difficult to accurately predict the actions of these kinds of therapeutic medications without undesirable side effects within the central nervous system (CNS) due to the complex interrelation of neuronal circuits^3,6,7^.

These difficulties led the researchers to develop pharmacokinetic approaches that target the drug itself. A pharmacokinetic approach aims to change the drug distribution or accelerating its clearance, thereby reducing or preventing its pharmacodynamic effect^8^. Protein-based pharmacokinetic approaches, including therapeutic enzymes, antibodies, and vaccines, are not expected to cross the blood–brain barrier (BBB) and, thus, are not expected to block normal functions of the CNS^9–12^. So, protein therapeutic approaches are attractive.

Further, within the pharmacokinetic approaches, an enzyme therapy has a major advantage over an antibody or vaccine approach. An enzyme does not require an immune response to be effective like the vaccine approach, and each enzyme molecule can degrade multiple drug molecules, which is dependent on the turnover number (*i.e*. catalytic rate constant *k*_cat_). In comparison, each antigen binding site of an antibody can only bind with one drug molecule and the antibody would be saturated at a high drug concentration in the body^12^. An ideal medication to treat cocaine use disorder would accelerate cocaine metabolism to produce biologically inactive metabolites by administration of an efficient cocaine-specific enzyme. Because an exogenous enzyme is not expected to cross BBB to affect the brain’s normal functions^10–13^, development of an efficient enzyme to specifically accelerate cocaine hydrolysis at the benzoyl ester^8,14–18^ has been recognized as a promising approach for treatment of cocaine use disorder.

It is known that cocaine has the same three metabolic pathways in both rats and humans^19–21^. First, cocaine is hydrolyzed mainly on the methyl ester group by a hepatic enzyme, known as human carboxylaterase-1 (hChE-1), to produce benzoylecgonine which is arguably biologically active^22–24^. Second, cocaine is hydrolyzed on the benzoyl ester group by a plasma enzyme, known as butyrylcholinesterase (BChE), to produce biologically inactive metabolites: ecgonine methyl ester (EME) and benzoic acid. In addition, another metabolite, norcocaine, is formed from cocaine oxidation catalyzed by liver microsomal cytochrome P450 (CYP) 3A4 (CYP3A4). Among these metabolites, norcocaine significantly contributes to the toxicity of cocaine, because it is more toxic than cocaine itself^25,26^. Therefore, the most desirable metabolic pathway for amplification is BChE-catalyzed cocaine hydrolysis on the benzoyl ester group. However, the catalytic efficiency of wild-type human BChE against naturally occurring (−)-cocaine is too low (*k*_cat_ = 4.1 min^−1^ and *K*_M_ = 4.5 μM)^27^ to be effective for treatment of cocaine overdose or dependence.

To improve the catalytic efficiency of human BChE against (−)-cocaine, we developed a structure and mechanism-based computational enzyme redesign approach^28–31^. The structure and mechanism-based computational enzyme redesign followed by experimental studies led to successful design and discovery of a series of new BChE mutants with considerably improved catalytic efficiency against (−)-cocaine and its toxic metabolites compared to the wild-type^32–36^. Particularly, our computationally designed BChE mutants with ≥ 1000-fold improved catalytic efficiency against cocaine compared to wild-type BChE^37–40^ are recognized as the *true* cocaine hydrolases (CocHs) in literature^41–45^. Our computationally designed, discovered, and patented first CocH, denoted as CocH1 (*i.e*. the A199S/S287G/A328W/Y332G mutant)^37,46–48^, has been truncated after amino-acid residue #529 and fused with human serum albumin (HSA or Albu)^41,49^ to extend its biological half-life for clinical development to treat cocaine dependence. This fusion protein is also known as Albu-CocH, Albu-CocH1, Albu-BChE, or TV-1380 in literature^41,50–54^. Extensive preclinical and clinical studies on TV-1380, including multiple Phase I clinical trials^50,51^ and a Phase II clinical trial^53^, favorably confirmed the safety of TV-1380 for its use in humans. According to the Phase II clinical trial^53^ with TV-1380 administered at a weekly dosing schedule, the enzyme significantly and dose-dependently decreased cocaine intake in the treatment groups compared to the placebo group, but its biological half-life (*t*_1/2_ = 8 h in rats and 43–77 h in humans) was not long enough for the practical cocaine dependence treatment using TV-1380 with the desired weekly dosing schedule.

In our previous studies, Fc-fused CocHs were constructed to further extend the half-life of CocHs and increase the CocH expression level^55–58^. Further, we have also developed stable cell lines that can stably express the Fc-fusion proteins in large scales^55,59,60^. The most efficient cocaine-metabolizing enzyme reported so far is CocH5 (the A199S/F227A/P285A/S287G/A328W/Y332G mutant of human BChE)^40^. To improve the half-life of the protein, a mutated Fc region (*i.e*. the A1V/M38Y/S40T/T42E/D142E/L144M mutant of Fc portion of human IgG1) was fused to CocH5 to generate a new protein entity known as CocH5-Fc(M6) (*k*_cat_ = 13,800 ± 750 min^−1^ and *K*_M_ = 3.89 ± 0.76 μM against (−)-cocaine at 37 °C)^60^. With a high specificity and a high catalytic efficiency against (−)-cocaine, CocH5-Fc(M6) can rapidly and selectively convert cocaine to non-toxic metabolites^61^. Meanwhile, the presence of the mutated Fc portion of the protein allows CocH5-Fc(M6) to bind with the neonatal Fc receptor (FcRn) under the acidic condition to achieve an unusually prolonged elimination half-life, with an elimination half-life of *t*_1/2_ = 229 ± 5 h in rats. It was demonstrated that intravenous (i.v.) administration of 1 mg/kg CocH5-Fc(M6) fully protected and rescued rats that had received the lethal dose of 180 mg/kg cocaine (i.p., LD_100_) by rapidly eliminating cocaine in blood stream for cocaine overdose treatment (note that the minimum LD_100_ = 100 mg/kg for cocaine i.p. in rats)^61^. It was also demonstrated that CocH5-Fc(M6) effectively blocked cocaine-induced dopamine transporter (DAT) trafficking (associated with cocaine dependence) with repeated cocaine exposures by maintaining a plasma CocH5-Fc(M6) concentration ≥ 58.7 ± 2.9 nM in rats^62,63^. Through protecting the dopaminergic system and allowing the system to recover from cocaine-induced DAT trafficking, CocH5-Fc(M6) has become a promising therapeutic candidate, suitable for treatment of cocaine dependence^60,62–64^. So, our further development of an enzyme therapy for treatment of cocaine use disorder has been focused on CocH5-Fc(M6). As a result of the effort, the high-level production of high-purity human recombinant CocH5-Fc(M6) protein in large-scale is available via Contract Research Organization (CRO) services at Catalent Pharma Solutions for the purpose of future clinical trials.

On the other hand, it is also known that development of a therapeutic protein for practical clinical use is usually a grand challenge, because a practically valuable therapeutic protein requires not only the desired functions of the protein, but also the stability associated with the therapeutic protein storage conditions and the corresponding shelf life. In fact, thermal stability of proteins is a common concern for most therapeutic protein development efforts. In general, the effectiveness of a drug product is affected by its stability. It is essential to attain the desired stability to ensure the quality of a therapeutic. Here we report for the first time the long-term thermal stability of CocH5-Fc(M6) at various storage temperatures.

In this study, we studied the thermal stability of CocH5-Fc(M6) in aqueous solution form produced in a 10L bioreactor via CRO service by Catalent. We are particularly interested in five different storage temperatures: − 80, − 20, 4, 25, and 37 °C because − 80 °C freezers are typically used in research labs, industry, or hospitals capable of protein production and/or storage; − 20 °C and 4 °C are the regular temperature settings for most fridges; 25 °C is close to the room temperature, and 37 °C is the normal human body temperature. As is well known, the physical and chemical properties as well as structure of proteins could be changed under different temperatures. So, we evaluated the time courses of protein appearance, pH, structural integrity, and catalytic activity against cocaine in vitro and in vivo of the CocH5-Fc(M6) bulk drug substance for its in vitro and in vivo stability after 18-month storage under various temperatures (− 80, − 20, 4, 25, or 37 °C). The data associated with these temperatures will guide future protein product storage, transportation and usage etc. All the data obtained suggest that CocH5-Fc(M6) has the properties desired for a therapeutic protein.

## Results

### Appearance

**Figure 1:**
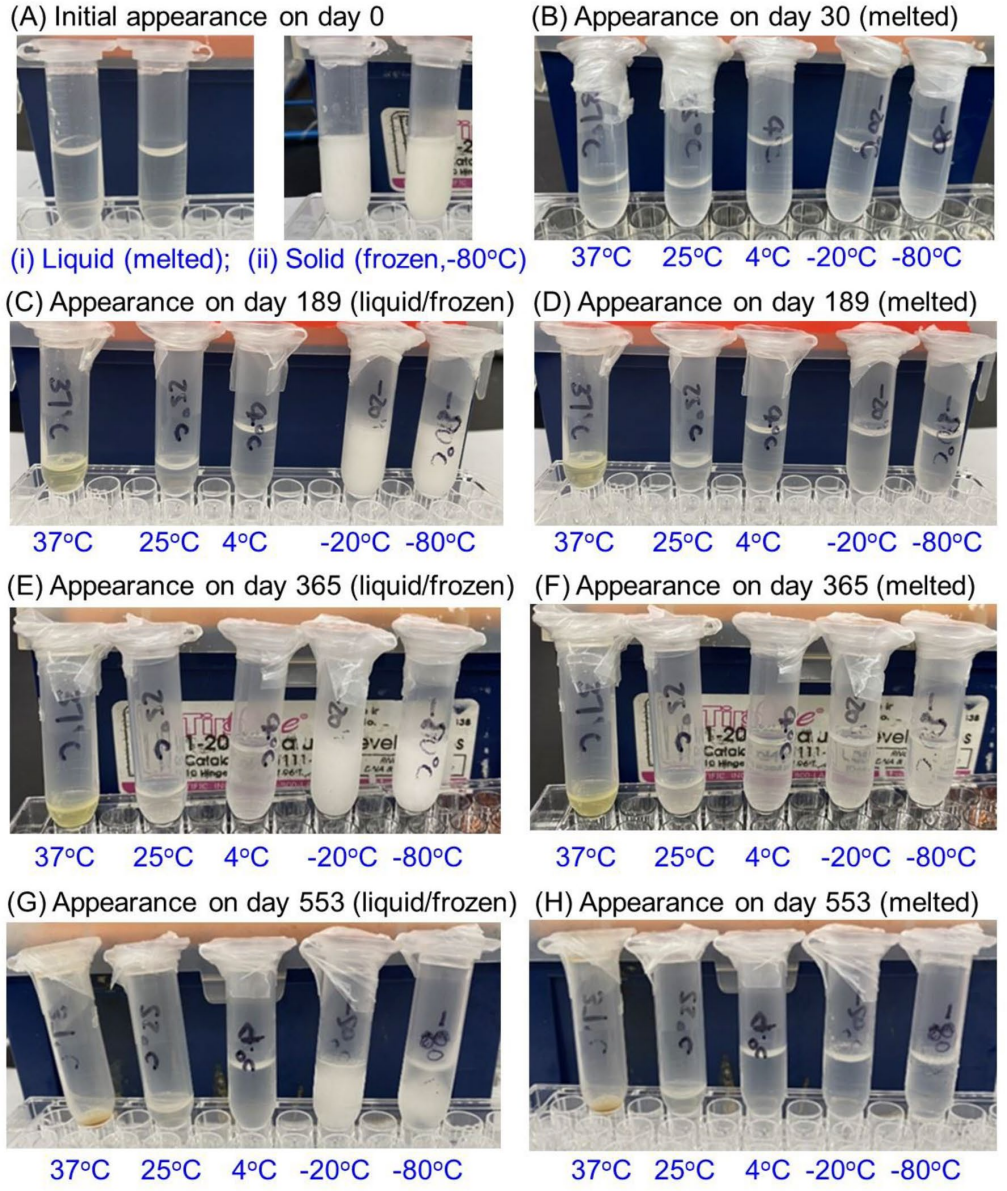
States of CocH5-Fc(M6) samples under different storage testing temperatures (37, 25, 4, − 20, and − 80 °C). (**A**) Initial appearance of samples at (i) liquid (melted) and (ii) solid (frozen) on day 0. (**B**) Liquid appearance after one month (day 30). (**C**) Appearance (liquid and solid) right out of storage place after ~ 6 months (day 189). (**D**) Appearance of liquid (melted) samples after ~ 6 months (day 189). (**E**) Appearance (liquid and solid) right out of storage place after 12 months (day 365). (**F**) Appearance of liquid (melted) after 12 months (day 365). (**G**) Appearance (liquid and solid) right out of storage place after ~ 18 months (day 553). (**H**) Appearance of liquid (melted) samples after ~ 18 months (day 553). For panels B to H, the five protein samples within each panel are in the order of 37, 25, 4, − 20, and − 80 °C from the left side to the right side.

A batch of the purified CocH5-Fc(M6) protein was produced by Catalent via CRO (Contract Research Organization) services. The CocH5-Fc(M6) protein was in a liquid formulation with purity of 99.59% and was shipped to our lab in a FedEx package with dry ice. As depicted in Fig. 1A, on day 0, the colors of frozen samples were observed white and the melted liquid samples were transparent. The stock solution was separated into multiple 2 ml centrifuge tubes stored under different temperatures, including − 80, − 20, 4, 25, and 37 °C.

As seen in Fig. 1B, after one month of the storage period (day 30), there was no significant appearance change for all five tubes of the enzyme samples stored under different temperatures. However, after ~ 6 months (day 189, Fig. 1C,D), the color of the enzyme sample stored under 37 °C changed from transparent to yellow and the color became even darker after 12 months (day 365, Fig. 1E,F) and ~ 18 months storage (day 553). Meanwhile, a slight color change for the CocH5-Fc(M6) sample stored at 25 °C was perceived after ~ 18 months storage (day 553) as shown in Fig. 1G,H. No notable changes in appearance were observed in any of the enzyme samples stored at − 80, − 20, or 4 °C on day 553. During the entire period of ~ 18 months, no precipitation or particle was observed.

### In vitro properties and stability of CocH5-Fc(M6)

**Figure 2:**
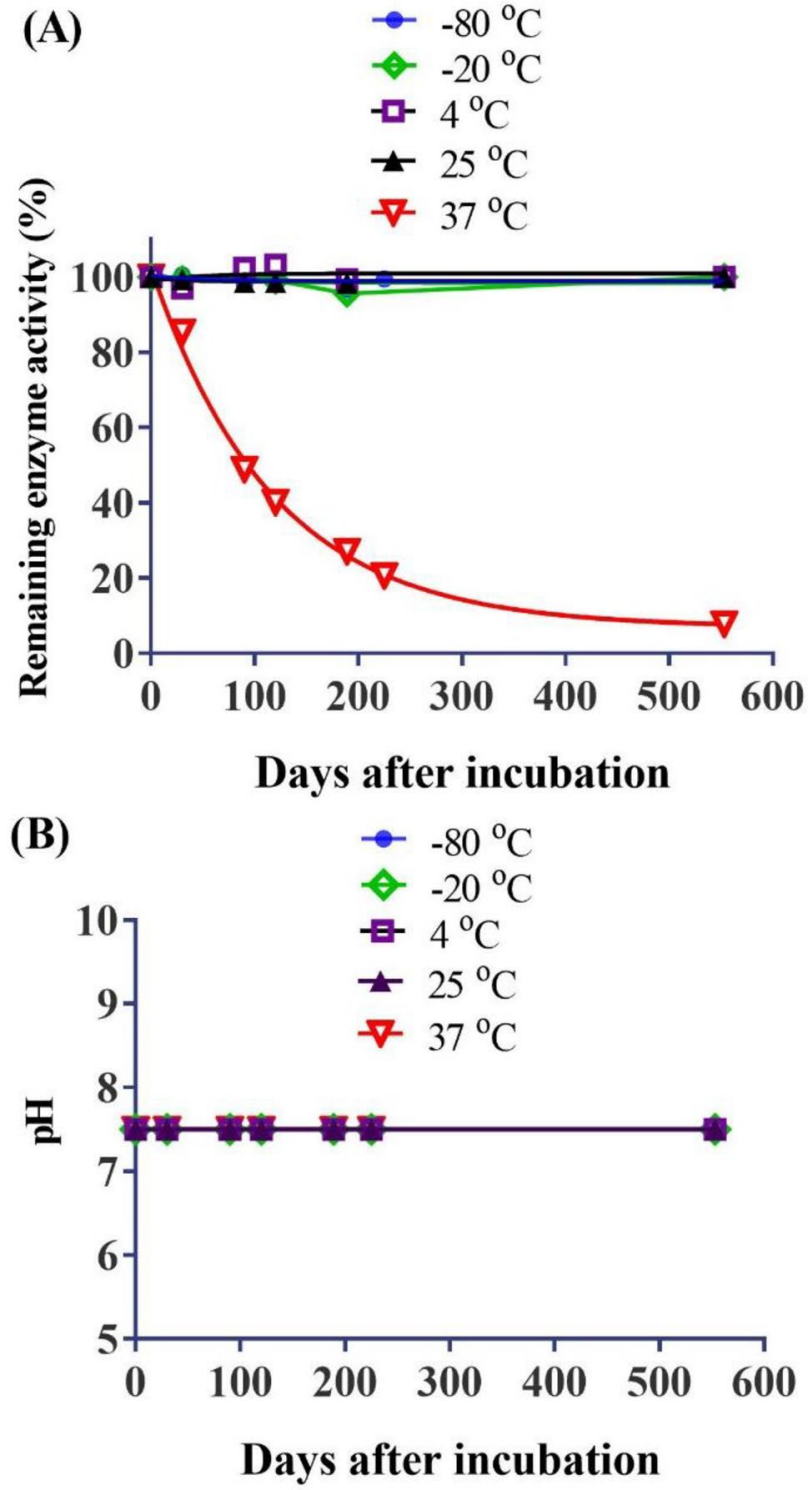
In vitro properties of the purified CocH5-Fc(M6) protein stored at various temperatures. (**A**) Remained in vitro enzyme activity against (−)-cocaine; (**B**) pH of the CocH5-Fc(M6) solution.

To characterize the in vitro properties of the CocH5-Fc(M6) protein and its long-time storage stability, we first determined its catalytic activity against (−)-cocaine at various time points from day 0 to day 553 after the enzyme samples were stored at various temperatures (− 80, − 20, 4, 25, and 37 °C). The obtained time-dependent enzyme activity data and pH are shown in Fig. 2. As shown in Fig. 2A, the in vitro enzyme activity against (−)-cocaine did not change significantly after the enzyme was incubated for 553 days at all the temperatures, except 37 °C. At 37 °C, CocH5-Fc(M6) protein had a limited stability profile, with a half-life of ~ 82 days. At any other temperatures (− 80, − 20, 4, or 25 °C), the in vitro enzyme activity of CocH5-Fc(M6) protein did not change at all over time within 553 days. Meanwhile, as seen in Fig. 2B, the pH of the enzyme solution did not change over time when it was stored at any of the temperatures (pH = 7.4 for all the measurements).

**Figure 3:**
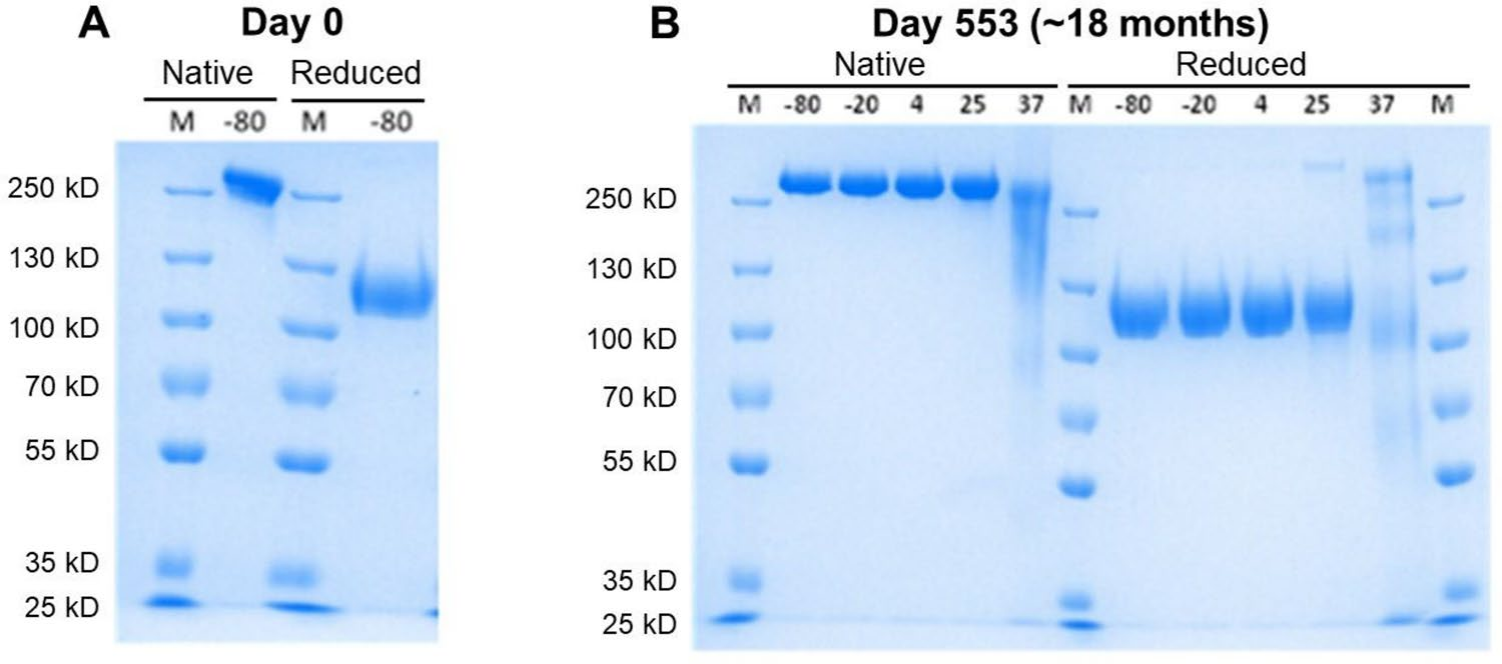
Native and reduced SDS-PAGE gels of the purified CocH5-Fc(M6) protein (a full-length gel for each panel, without grouping). (**A**) Initially received protein (day 0). (**B**) Day 553 (~ 18 months) after the protein was stored at various temperatures: − 80 °C (indicated as − 80), − 20 °C (− 20), 4 °C (4), 25 °C (25), and 37 °C (37). The markers were indicated as M.

Further, the purified CocH5-Fc(M6) protein was analyzed using SDS-PAGE electrophoresis on days 0 and 553 of the incubation under different temperatures. Specifically, on day 0, we first applied non-reducing (left)/reducing (right) SDS-PAGE to characterize the protein (Fig. 3 and also Fig. S1 in Supplementary Information). As shown in Fig. 3A, the intact CocH5-Fc(M6) existed as a dimer and had a size (molecular weight) of ~ 250 kDa. Under the reducing condition, the disulfide bonds in CocH5-Fc(M6) were broken down and reduced to the monomer with a molecular weight being close to ~ 130 kDa. The purity of CocH5-Fc(M6) was confirmed by the SDS-PAGE data obtained on day 0 (Fig. 3A).

After ~ 18 months (day 553) of storage (Fig. 3B), the CocH5-Fc(M6) protein stored at − 80, − 20, 4, or 25 °C remained as a dimer in the native SDS-PAGE gel, and the reduced SDS-PAGE gel showed the expected monomer. So, the dimer structure did not change over time for the protein stored at any of these temperatures (− 80, − 20, 4, and 25 °C), while the sample under the 25 °C storage showed relatively more impurity with lower/higher molecule weights, compared to other samples.

Notably, after ~ 18 months (day 553) of the incubation at 37 °C, the CocH5-Fc(M6) protein still mainly existed in the dimer, but significant portion of the protein had lower molecular weights, which suggests the protein degradation and explains why the in vitro enzyme activity considerably decreased on day 553 of the incubation at 37 °C. The reduced SDS-PAGE gel of the protein stored at 37 °C for ~ 18 months (day 553) is consistent with the degradation and structural change of the CocH5-Fc(M6) dimer (Fig. 1 and Fig. S1 in Supplementary Information).

### In vivo activity and stability of CocH5-Fc(M6)

#### Ex vivo cocaine hydrolysis activity and pharmacokinetics in rats

**Figure 4:**
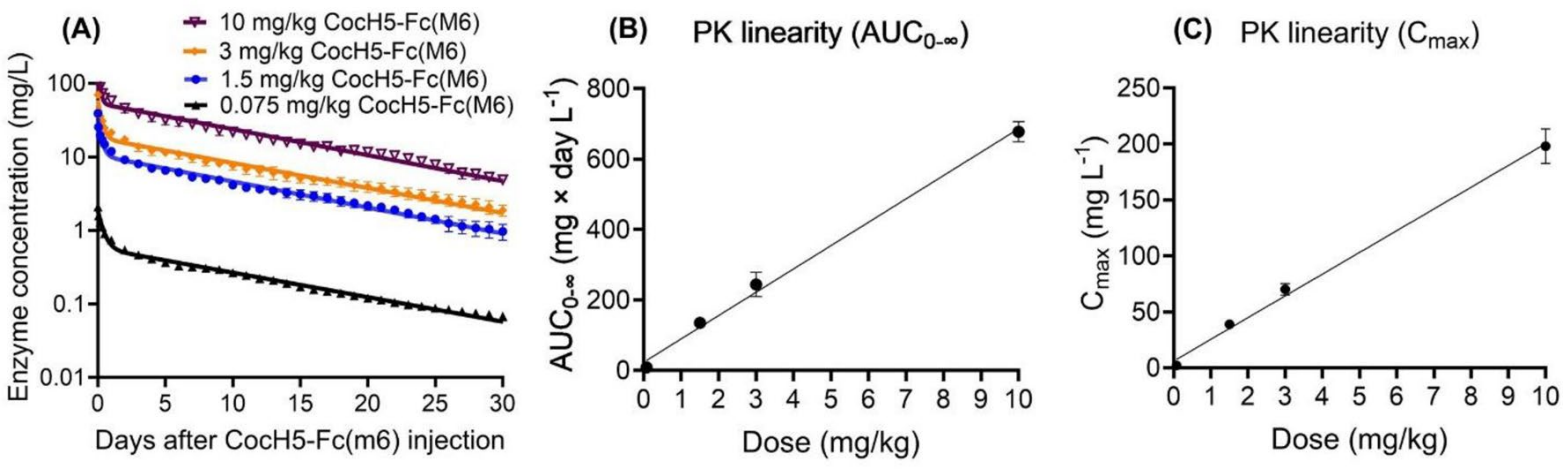
PK profile of CocH5-Fc(M6) in rats (n = 3 per group) received i.v. administration of 0.075, 1.5, 3 or 10 mg/kg CocH5-Fc(M6). (**A**) Time-dependent plasma concentrations of CocH5-Fc(M6). (**B**) Dose dependence of AUC_0-∞_, showing the linearity of the PK profile. (**C**) Dose dependence of C_max_, showing the linearity of the PK profile. The data are shown as Mean ± SEM.

Knowing the in vitro properties and stability of CocH5-Fc(M6), we wanted to further determine its in vivo activity and stability. For the in vivo activity, we first determined the cocaine hydrolysis activity and the corresponding pharmacokinetic (PK) profile of the CocH5-Fc(M6) after intravenous (i.v.) administration of the enzyme at various doses (0.075, 1.5, 3 and 10 mg/kg) in rats within first 3 months after receiving the protein material. Figure 4A depicts the time-dependent plasma concentrations of CocH5-Fc(M6) after the enzyme administration. Summarized in Table 1 are the PK parameters obtained from the non-compartment analysis. All these data consistently revealed that the CocH5-Fc(M6) protein followed the linear elimination pattern, with the clearance in the range of ~ 8.65–14.8 mL/day/kg and with an elimination half-life of ~ 9 days. The AUC (area under the curve) and the maximum concentration (*C*_max_) were linearly dependent on the dose as shown in Fig. 4B,C. The volume of distribution based on the terminal phase (Vz) and the estimate of volume of distribution at steady-state based on the last observed non-steady-state data (Vss) varied from 113.8–198.1 to 108.7–186.7 mL/kg, respectively, corresponding to CocH5-Fc(M6) doses of 0.075–10 mg/kg, while mean residence time from the time of dosing to the time of the last measurable concentration (MRT_0-t_) (~ 9 days) and mean residence time from the time of dosing to the infinite (MRT_0-∞_) (~ 12 days) basically remain constants for all the enzyme doses. These PK parameters of CocH5-Fc(M6) produced by Catalent are consistent with our previous PK data obtained by using the CocH5-Fc(M6) protein material produced in our lab^60^.

**Table 1:** Pharmacokinetic parameters of CocH5-Fc(6M) by the non-compartmental analysis.

Dose (mg/kg, i.v.)	0.075	1.5	3	10
C_max_ (mg L^−1^)^a^	2.17 ± 0.09	38.92 ± 1.64	70.17 ± 5.10	198.02 ± 15.41
t_1/2_ (day)	9.11 ± 0.31	8.75 ± 0.82	9.38 ± 0.41	9.26 ± 0.47
CL (mL × day^−1^ kg^−1^)^b^	8.65 ± 0.70	11.29 ± 1.15	12.7 ± 1.67	14.8 ± 0.67
Vz (mL/kg)^c^	113.81 ± 9.05	139.91 ± 0.27	171.04 ± 17.80	198.09 ± 13.76
Vss (mL/kg)^d^	108.72 ± 8.97	132.82 ± 2.11	160.15 ± 16.30	186.72 ± 11.64
MRT_0-t_ (day)^e^	8.95 ± 0.16	8.93 ± 0.48	9.12 ± 0.23	9.29 ± 0.28
MRT_0-∞_ (day)^f^	12.58 ± 0.55	12.02 ± 1.27	12.68 ± 0.52	12.60 ± 0.56
AUC_0-24_ (mg × day L^−1^)	1.11 ± 0.07	16.55 ± 0.36	28.99 ± 2.95	75.28 ± 3.47
AUC_0-t_ (mg × day L^−1^)	7.91 ± 0.69	122.56 ± 15.07	218..76 ± 29.55	612.01 ± 26.93
AUC_0-∞_ (mg × day L^−1^)	8.85 ± 0.74	135.37 ± 12.52	244.44 ± 34.60	677.69 ± 28.94

^a^*C*_max_—Maximum observed concentration.

^b^*CL*—Clearance.

^c^*V*_*Z*_—Volume of distribution based on the terminal phase.

^d^*V*_*SS*_—An estimate of the volume of distribution at steady-state based on the last observed non-steady-state data.

^e^*MRT*_*0-t*_—Mean residence time from the time of dosing to the time of the last measurable concentration.

^f^*MRT*_*0-∞*_—Mean residence time from the time of dosing to the infinite.

#### In vivo stability of CocH5-Fc(M6)

**Figure 5:**
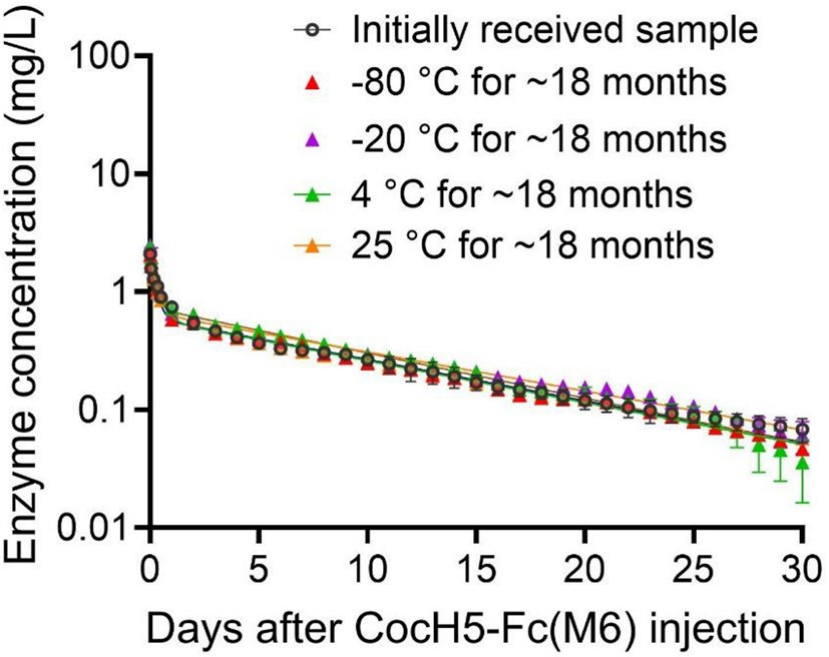
Time-dependent plasma concentrations of CocH5-Fc(M6) in rats (n = 3 per group) received i.v. administration of 0.075 of CocH5-Fc(M6) after it was stored at various temperatures (− 80 °C, − 20 °C, 4 °C, and 25 °C) for ~ 18 months. The data are shown as Mean ± SEM. The PK data at the same dose of 0.075 mg/kg for the initially received CocH5-Fc(M6) sample (the day 0 reference shown in Fig. 4) is also included in this figure for comparison. There was no significant difference in the normalized enzyme concentrations between any of these CocH5-Fc(M6) protein samples after ~ 18 months and the initially received CocH5-Fc(M6) protein sample according to the one-way ANOVA (*p* = 0.9992 for − 80 °C, *p* = 0.6025 for − 20 °C, *p* = 0.7503 for 4 °C, and *p* = 0.9998 for 25 °C).

In order to examine the stability of the in vivo activity of the enzyme, after the purified CocH5-Fc(M6) protein was stored at various temperatures (− 80 °C, − 20 °C, 4 °C, and 25 °C) for ~ 18 months (553 days), the enzyme samples were administered (i.v.) to rats at a dose of 0.075 mg/kg and plasma enzyme concentrations were determined at various time points within 30 days after the enzyme administration. Depicted in Fig. 5 are the time-dependent plasma concentrations of CocH5-Fc(M6) in rats after the protein samples were stored for ~ 18 months compared to the initially received protein sample (the day 0 reference).

As shown in Fig. 5, the time-concentration profiles of the enzyme samples stored under four different temperatures are very similar to each other. The PK parameters were further analyzed by the non-compartment model, showing no significant difference in these parameters between the initially received CocH5-Fc(M6) protein sample and the protein samples after ~ 18 months of the storage at any of the four different temperatures. Summarized in Table 2 are the PK parameters of the CocH5-Fc(M6) protein samples stored at four different temperatures (− 80 °C, − 20 °C, 4 °C, and 25 °C) for ~ 18 months in comparison with the PK parameters determined for the initially received CocH5-Fc(M6) sample (the day 0 reference). Acording to the data in Table 2, the PK parameters of the CocH5-Fc(M6) protein stored at any of these four temperatures (− 80 °C, − 20 °C, 4 °C, and 25 °C) for ~ 18 months are not significantly different from the corresponding PK parameters of the initially received CocH5-Fc(M6) sample.

**Table 2:** PK parameters determined by the non-compartment analysis for the CocH5-Fc(M6) protein samples stored at various temperatures (− 80 °C, − 20 °C, 4 °C, and 25 °C) for ~ 18 months in comparison with the PK parameters determined for the initially received CocH5-Fc(M6) sample.

Dose (0.075 mg/kg, i.v.)	Day 0 (ref)	18 Months (− 80 °C)	*p*	18 Months (− 20 °C)	*p*	18 Months (4 °C)	*p*	18 Months (25 °C)	*p*
C_max_ (mg L^−1^)	2.17 ± 0.09	2.06 ± 0.12	1.0	2.50 ± 0.31	0.13	2.40 ± 0.03	0.30	2.34 ± 0.08	0.46
t_1/2_ (day)	9.11 ± 0.31	8.51 ± 0.11	0.70	9.13 ± 0.94	1.0	8.29 ± 0.72	0.51	9.05 ± 0.02	1.0
CL (mL × day^−1^ kg^−1^)	8.65 ± 0.70	9.78 ± 0.09	0.81	8.20 ± 0.25	0.40	8.88 ± 0.71	0.96	9.56 ± 0.58	0.96
Vz (mL/kg)	113.81 ± 9.05	105.8 ± 16.87	0.93	108.30 ± 7.71	0.98	104.72 ± 1.51	0.89	124.77 ± 7.47	0.99
Vss (mL/kg)	108.72 ± 8.97	102.4 ± 9.72	0.95	101.48 ± 9.71	0.91	89.74 ± 5.12	0.30	116.05 ± 5.79	0.91
MRT_0-t_ (day)	8.95 ± 0.16	8.61 ± 0.20	0.72	9.21 ± 0.28	0.87	8.60 ± 0.45	0.71	8.94 ± 0.12	0.99
MRT_0-∞_ (day)	12.58 ± 0.55	10.35 ± 1.01	0.29	12.44 ± 1.49	0.99	10.30 ± 1.35	0.27	12.17 ± 0.14	0.99
AUC_0-24_ (mg × day L^−1^)	1.11 ± 0.07	1.03 ± 0.03	0.77	1.11 ± 0.09	0.99	1.09 ± 0.02	0.99	1.01 ± 0.04	0.48
AUC_0-t_ (mg × day L^−1^)	7.91 ± 0.69	7.17 ± 0.16	0.66	8.26 ± 0.18	0.96	8.09 ± 0.47	0.99	7.14 ± 0.45	0.62
AUC_0-∞_ (mg × day L^−1^)	8.85 ± 0.74	7.66 ± 0.07	0.82	9.10 ± 0.38	0.45	8.55 ± 0.70	0.94	7.89 ± 0.50	0.97

All the PK parameters were based on the i.v. administration of 0.075 mg/kg enzyme samples (n = 3/group). Statistical analysis: *p* refers to the comparison with the day 0 reference using the one-way ANOVA analysis.

## Discussion

In the lack of an FDA-approved treatment specific for cocaine dependence or overdose, development of an efficient enzyme to specifically accelerate cocaine hydrolysis at the benzoyl ester^8,14–18^ is recognized as a promising approach for treatment of cocaine use disorder. CocH5-Fc(M6) (a BChE mutant fused to a mutant of Fc in human IgG1) is the currently known most efficient long-acting cocaine hydrolase with both the highest catalytic activity (*k*_cat_ = 13,800 ± 750 min^−1^, *K*_M_ = 3.89 ± 0.76 μM) against (−)-cocaine and the longest biological half-life (*t*_1/2_ = 229 ± 5 h) in rats. In the current study, we characterized the CocH5-Fc(M6) protein produced in a 10-L bioreactor by Catalent for its in vitro and in vivo profiles as well as their stability after long-time storage at various temperatures. According to the time-dependent in vitro properties of the protein after its storage at − 80, − 20, 4, 25, or 37 °C for ~ 18 months, the CocH5-Fc(M6) protein was very stable at any of the four temperatures (− 80, − 20, 4, and 25 °C), without significant changes in the properties, the catalytic activity against (−)-cocaine and the pH. In comparison, at 37 °C, the protein was less stable, with a half-life of ~ 82 days for the activity against cocaine. Furthermore, we examined the in vivo profiles of the CocH5-Fc(M6) protein after it was stored at − 80, − 20, 4, or 25 °C for ~ 18 months in comparison with the corresponding in vivo profile of the originally received protein sample (the day 0 reference). Based on the in vivo data obtained, the in vivo profiles of the CocH5-Fc(M6) protein were very stable at any of the four temperatures (− 80, − 20, 4, and 25 °C) for ~ 18 months, without significant changes in the in vivo profiles. The favorable in vitro and in vivo stability of the CocH5-Fc(M6) protein suggests that the protein product may be stored simply in room temperature or 4 °C (a more conservative choice). Additionally, for the in vivo profiles obtained, we have determined the PK profiles of CocH5-Fc(M6) at four different dose levels (from 0.075 to 10 mg/kg, with a wider range in which the highest dose is > 100-fold higher than the lowest dose), which confirms the linear PK profile for CocH5-Fc(M6) with an elimination half-life (*t*_1/2_) of ~ 9 days. All these in vitro and in vivo data on the stability and efficacy of CocH5-Fc(M6) consistently suggest that CocH5-Fc(M6) indeed has the desired in vitro and in vivo stability as a promising enzyme therapy candidate for treatment of cocaine use disorder.

The observed long-term stability of CocH5-Fc(M6) stored at 4 and 25 °C is particularly interesting because a protein drug stored at 4 or 25 °C can be used for administration immediately in emergency situations such as cocaine overdose, whereas a frozen drug must be thawed before it can be administered and, hence, has a delayed time for use. Like TV-1380 (an albumin fused CocH1 tested in Phase I & II clinical trials for cocaine addiction treatment), a more convenient route such as intramuscular (i.m.) suitable for self-administration by patients, is also available for CocH5-Fc(M6) which will be addressed in subsequent preclinical studies.

Finally, the obtained results in this investigation are consistent with the previously known thermal stability of the relevant protein—wild-type human BChE, which was found to be stable when it was stored in a lyophilized form over years and was proven to be safe and non-toxic at a dose that is 30 times higher than the therapeutic dose in mice by general observation, serum chemistry, hematology, gross or histologic tissue changes^65^. Similarly, no mice died by i.v. administration of 100 mg/kg CocH5-Fc(M6) in our preliminary toxicity testing (data not shown). The doses of CocH5-Fc(M6) used in this study did not cause any mortality to rats signaling its safety as a therapeutic candidate, although the formal toxicity studies on CocH5-Fc(M6) need to be carried out under the FDA-required good laboratory practice (GLP).

## Materials and methods

### Materials

Purified CocH5-Fc(M6) protein bulk drug substance (BDS) was produced by Catalent (Madison, WI) using Catalent’s GPEx^®^ technology through CRO (Contract Research Organization) services. Using the GPEx^®^ technology, a stable Chinese Hamster Ovary (CHO) cell line expressing CocH5-Fc(M6) was first developed, a master cell bank (MCB) was generated, and a purification protocol was established. The purified CocH5-Fc(M6) protein produced at Catalent by using the MCB cells and a 10-L bioreactor in 2021 was received on 6/29/2021 as day 0. (−)-Cocaine was provided by the National Institute on Drug Abuse (NIDA) Drug Supply Program (Bethesda, MD). [^3^H](−)-cocaine (50 Ci/ mmol) was ordered from PerkinElmer (Waltham, Massachusetts). All other general chemicals and supplies were purchased from Thermo Fisher Scientific (Waltham, MA), Sigma-Aldrich (St. Louis, MO), or VWR International (Radnor, PA).

### Animals

Male Sprague–Dawley rats (250–275 g) used in this study were ordered from Harlan/Envigo (Indianapolis, IN), and housed initially as one or two rats per cage. All the in vivo experiments were carried out in our animal lab within the animal laboratories of the Division of Laboratory Animal Resources (DLAR) facility (PHS assurance number A3336-01; USDA number 61-R-0002; AAALAC, Intl. Unit # 13) at the University of Kentucky, with the DLAR staff providing veterinary care and animal husbandry services. All rats were allowed ad libitum access to food and water and maintained on a 12 h light/12 h dark cycle, with the lights on at 8:00 a.m. at a room temperature of 21–22 °C. Experiments were performed in the same colony room in accordance with the Guide for the Care and Use of Laboratory Animals as adopted and promulgated by the National Institutes of Health, and were in fact also consistent with the ARRIVE (Animal Research: Reporting of In Vivo Experiments) guidelines (https://arriveguidelines.org). The animal protocol was approved by the IACUC (Institutional Animal Care and Use Committee) at the University of Kentucky.

### In vitro stability of CocH5-Fc(M6)

Aliquots of CocH5-Fc(M6) were stored at five different temperatures: − 80, − 20, 4, 25, and 37 °C in a VWR freezer (− 80 °C), a Frigidaire refrigerator (− 20 °C and 4 °C), a lab cabin (22–25 °C) and a Fisherbrand basic 60-L gravity incubator (37 °C). Samples were evaluated on day 0, day 30 (1 month), day 189 (~ 6 months), day 365 (12 months), and day 553 (~ 18 months) after the protein was stored under different temperatures. The appearance of CocH5-Fc(M6) was measured by general (visual) observation and pH was measured by using pH testing strips.

The enzyme activity of the CocH5-Fc(M6) protein was analyzed by a sensitive radiometric assay based on toluene extraction of [^3^H](−)-cocaine labeled on its benzene ring^38^. The enzymatic reactions proceeded at room temperature (25 °C), to initiate the enzymatic reaction. Specifically, 50 μl of 400 μM [^3^H](−)-cocaine was mixed with 140 μl of 0.1 M sodium phosphate buffer and 10 μl of CocH5-Fc(M6) solution. The reactions were stopped by adding 200 μl of 0.1 N HCl. [^3^H]benzoic acid was extracted by 1 ml of toluene and measured by scintillation counting. Finally, the measured (−)-cocaine concentration-dependent radiometric data were analyzed by using the standard Michaelis–Menten kinetics with the known catalytic parameters (catalytic rate constant *k*_cat_ and Michaelis–Menten constant *K*_M_) to determine the actual active enzyme activity.

The purified CocH5-Fc(M6) protein was analyzed by sodium dodecyl sulphate–polyacrylamide gel electrophoresis (SDS-PAGE) electrophoresis. Protein samples were prepared in non-reducing or reducing sample loading buffers (Thermo Fisher Scientific, Waltham, MA). The protein samples that were mixed with reducing loading buffer were heated at 95 °C for 5 min. All the protein samples were loaded in a 4–12% Tris–Glycine Mini Protein Gel, and 100 voltage was applied for 2 h. The protein gel was stained with the SimpleBlue SafeStain for 1 h and washed in water for 3 h to be ready for photography.

### In vivo activity and stability of CocH5-Fc(M6)

In vivo circulatory stability of the enzyme was determined by measuring the ex vivo cocaine hydrolysis activity and the corresponding pharmacokinetic profile of the enzyme samples (after they were stored at different temperatures for various time durations) after the i.v. administration in rats. On day 0 and within the first three months after receiving the enzyme, a dose of 0.075, 1.5, 3, or 10 mg/kg CocH5-Fc(M6) (in vehicle of 1 M glycine, 20% (w/v) sorbitol, and 0.05 M HEPES at pH 7.4) was injected in rats (n = 3/group) via tail veins. After the i.v. injection, blood samples of about 100 μl were collected into heparinized centrifuge tubes from the saphenous veins at various time points. Anesthesia was not used for all the i.v. administrations and blood collections. Then the blood samples were centrifuged at 5000 g for 15 min and the separated plasma samples were kept at 4 °C before analysis. Similar in vivo tests were also performed for the protein samples stored at various temperatures after ~ 18 months, with rats (n = 3/group) injected (i.v.) with 0.075 mg/kg of CocH5-Fc(M6) via tail veins on day 575. A radiometric assay using 100 μM (−)-cocaine was performed to measure the ex vivo cocaine hydrolysis activity of the enzyme in the collected plasma samples^55,57^.

The measured time-dependent ex vivo cocaine hydrolysis activity data were fitted to a double-exponential equation ([E]_*t*_ = A*e*^−k1*t*^ + B*e*^−k2*t*^) by using the GraphPad Prism 9 software (GraphPad Software Inc., San Diego, CA).

### Data analysis

All animal data were analyzed by using the GraphPad Prism 7 software (GraphPad Software, La Jolla, CA). Data are presented as mean ± SEM. In addition, the Phoenix WinNonlin software (Certara, Princeton, NJ) was used for non-compartment analysis of the PK data. The PK parameters obtained on day 0 were used as the reference standard for comparing with the corresponding PK profiles after ~ 18 months of storage at different temperatures with one-way analysis of variance (ANOVA). A difference was considered significant when the *p* < 0.05.

### Supplementary Information

Supplementary Figure S1.

## Data Availability

The datasets used and/or analyzed during the current study are available from the corresponding author on reasonable request.
