# Supplementary material for: In vitro and in vivo stability of a highly efficient long-acting cocaine hydrolase
Source: Sci Rep. 2024 May 13;14:10952. doi: 10.1038/s41598-024-61646-7 (PMC11091111; doi:10.1038/s41598-024-61646-7)
Supplement: Supplementary file 1 — Supplementary Figure S1. [file 41598_2024_61646_MOESM1_ESM.pdf]

<u>Supplementary Materials</u>

In vitro and *in vivo* stability of a highly efficient long-acting cocaine hydrolase

Linyue Shang[1,2,†], Huimei Wei[1,2,†], Jing Deng[1,2,†], Madeline J. Stewart[1,2], Johnathan E. LeSaint[1,2], Annet Kyomuhangi[1,2], Shawn Park[1,2], Elise C. Maul[1,2], Chang-Guo Zhan[1,2,*], and Fang Zheng[1,2,*]

[1]*Molecular Modeling and Biopharmaceutical Center and* [2]*Department of Pharmaceutical Sciences, College of Pharmacy, University of Kentucky, 789 South Limestone Street, Lexington, KY 40536*

Running Title: Stability of a highly efficient cocaine hydrolase

[†] These authors contributed equally to this work.

* Corresponding authors. Email: zhan@uky.edu (C.-G.Z.) and fzhen2@uky.edu (F.Z.)

<u>Correspondence to</u>:
Chang-Guo Zhan, Ph.D.
Director, Molecular Modeling and Biopharmaceutical Center (MMBC)
Director, Chemoinformatics and Drug Design Core of CPRI
University Research Professor
Endowed College of Pharmacy Professor in Pharmaceutical Sciences
Professor, Department of Pharmaceutical Sciences
College of Pharmacy
University of Kentucky
789 South Limestone Street
Lexington, KY 40536
Phone: 859-323-3943
FAX: 859-257-7585
E-mail: zhan@uky.edu

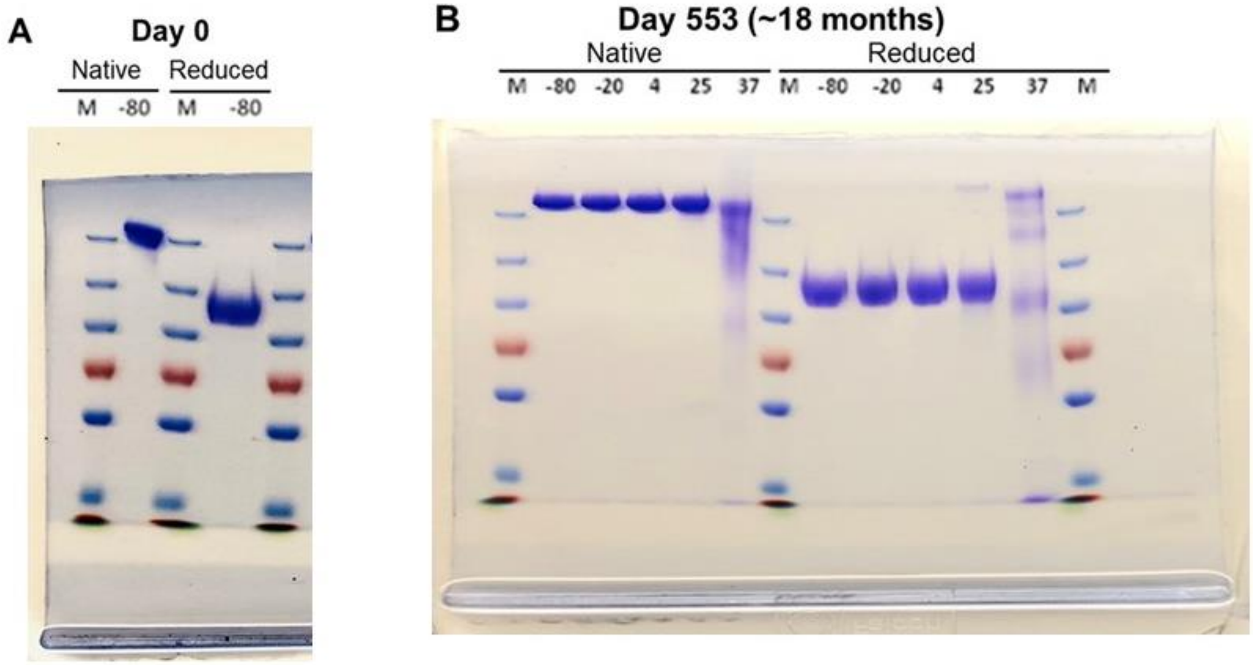

Figure S1. The original full-length blots for Figure 3 (Panels A and B). The protein gel was stained with the SimplyBlue SafeStain solution. SimplyBlue SafeStain is a ready-to-use, fast, sensitive, and safe Coomassie G-250 stain for visualizing protein bands on polyacrylamide gels. There is no hybridization (no immunoblotting) with antibodies during blotting. The gels after finishing electrophoresis were directly stained with the staining solution. No cut for the gel itself was performed. The loading wells were not stained.
